# How Fast Do Objects Fall in Visual Memory? Uncovering the Temporal and Spatial Features of Representational Gravity

**DOI:** 10.1371/journal.pone.0148953

**Published:** 2016-02-24

**Authors:** Nuno De Sá Teixeira

**Affiliations:** Institute of Cognitive Psychology–University of Coimbra, Coimbra, Portugal; Center for BrainHealth, University of Texas at Dallas, UNITED STATES

## Abstract

Visual memory for the spatial location where a moving target vanishes has been found to be systematically displaced downward in the direction of gravity. Moreover, it was recently reported that the magnitude of the downward error increases steadily with increasing retention intervals imposed after object’s offset and before observers are allowed to perform the spatial localization task, in a pattern where the remembered vanishing location drifts downward as if following a falling trajectory. This outcome was taken to reflect the dynamics of a representational model of earth’s gravity. The present study aims to establish the spatial and temporal features of this downward drift by taking into account the dynamics of the motor response. The obtained results show that the memory for the last location of the target drifts downward with time, thus replicating previous results. Moreover, the time taken for completion of the behavioural localization movements seems to add to the imposed retention intervals in determining the temporal frame during which the visual memory is updated. Overall, it is reported that the representation of spatial location drifts downward by about 3 pixels for each two-fold increase of time until response. The outcomes are discussed in relation to a predictive internal model of gravity which outputs an on-line spatial update of remembered objects’ location.

## Introduction

Given the inherently noisy functioning of sensory processing and as neural pathways relay information with sizeable delays [1, 2], living creatures are faced with an important predicament–in order to be attuned to the world dynamics, some degree of prediction and anticipation in perceptual functions is required for meaningful and coordinated interactions. By virtue of its ubiquity, gravity ought to be one of the most powerful of such ecological invariants, structuring and constraining virtually any interaction with and within any given environment. Accordingly, several research lines have come to acknowledge, during the last few decades, a role of an internal model of gravity, thought as a neural structure that explicitly mimics its mechanics, in a range of perceptual phenomena [3, 4, 5, 6, 7].

One such lines of enquiry focused on spatial distortions of remembered locations of moving objects. When shown a moving object that disappears unexpectedly and if required to locate, as precisely as possible, that vanishing position, human observers systematically report a point biased forward, in the direction of motion–*representational momentum*–and downward, in the direction of gravity–*representational gravity* [8].

Representational momentum, first reported with implied motion displays and a *same-different* judgment task [9], was originally interpreted as reflecting a cognitive analogue of physical momentum. In line with this reasoning, faster implied movements lead to an increase in its magnitude [10]. Moreover, representational momentum was found to increase with time until a peak at about 300 milliseconds [11]. In subsequent research, representational momentum was reported with smoothly moving objects and a behavioural localization task, where participants were required to position a mouse cursor on the remembered vanishing location onscreen [12]. Besides replicating previous outcomes (e.g., increased magnitude for faster moving objects), the behavioural localization paradigm revealed a set of additional effects which were invariably taken as reflecting cognitive analogues of various physical variables [8]. However, and as both smoothly moving objects and behavioural localization responses engage extraneous perceptual factors that, more often than not, were left uncontrolled in those studies, some doubts were raised regarding the degree to which some of those phenomena were in fact due to cognitive analogues of physical properties [13]. Thus, for instance, the displacement forward in the direction of motion (representational momentum) for smoothly moving targets was argued to be due to smooth pursuit eye movements which overshoot the object’s vanishing position coupled with the known foveal bias effect–proneness to locate targets as displaced toward the direction of gaze [14, 15]. In fact, when eye movements are constrained, representational momentum tends to be compromised [16] and spatial errors are made mostly in the direction of gaze, not motion. Additionally, preventing eye movements eliminates the effect of object’s velocity on the spatial mislocalizations [17, 18]. Similarly, whereas an inward displacement for the vanishing position of circularly moving targets was first interpreted as reflecting a naïve notion of centripetal impetus [19], further research showed that eye movements were drawn to the centre of the display in those conditions [20]. Target’s size, which was found to result in an increased displacement forward in the direction of motion [21] and downward in the direction of gravity [22, 23], in line with the hypothesis that observers judged bigger objects as heavier, was recently found to be due to an increased foveal bias when locating targets with larger spatial extents [17]. Similar accounts based on the coupling of eye movement patterns and foveal and spatial biases were put forth regarding the alleged sensitivity of representational momentum to the perception of causality [24, 25] and to implied friction [13, 14, 22, 25].

Human observers also systematically remember object’s vanishing locations as displaced downward in the direction of gravity–*representational gravity* [12]. This trend emerges both for targets moving horizontally, with a mnesic spatial error made downward in a direction orthogonal to motion, and vertically, where a bigger representational momentum is found for descending as compared to ascending objects. What is more, in a series of recent studies, it was reported that the displacement downward in the direction of gravity increases with time, up until at least 1200 to 1400 milliseconds, in a pattern where the remembered vanishing location drifts downward as if following an anticipated course–*representational trajectory–*taking into account an internal model of earth’s gravity [26, 27, 28]. Of relevance, these trends were found to be unrelated with the presence or absence of smooth pursuit eye movements, as constraining the observers’ gaze did not prevent the downwards drift of the remembered vanishing location.

**Fig 1:**
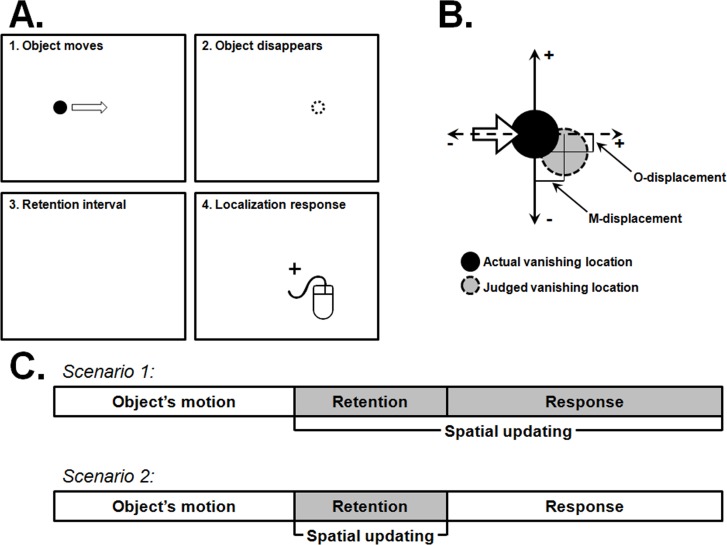
Panel A: Standard trial of a behavioural spatial localization task; Panel B–Measurement of spatial localization errors: M-displacement stands for the error made along the axis of motion (dashed axis) and O-displacement to the error orthogonal to the direction of motion (continuous axis); Panel C–Two scenarios regarding the temporal window of the spatial updating of the remembered vanishing location.

Notwithstanding, the specific spatial and temporal dynamics of representational trajectory are still to be ascertained. In previous research, the rate of the drift downward in the direction of gravity has been reported to be between 2 and 7.5 pixels per second [26, 28]. However, these estimates were done based only upon the imposed retention intervals after the offset of the moving object and did not take into account the time it took the participants to respond. Although some evidence has been reported that the magnitude of the displacement downward in the direction of gravity is not correlated with response times [17, 27], the issue is still largely unexplored [29]. To appreciate the point, consider a standard trial (see Fig 1, panel A): the observer is first shown a moving object that disappears unexpectedly at a certain point of its course. Immediately afterwards, a retention interval is imposed during which the observer has to retain the remembered vanishing location–at this stage, allegedly, the spatial memory is being updated with an influence of an internal model of gravity, thus accounting for the downward mnesic displacement. Upon the end of the retention, the observer must displace a cursor, using a computer mouse, to the position where he/she remembers seeing the object disappearing. Errors made along the axis of motion–M-displacement–and along the axis orthogonal to motion–O-displacement–are measured (see Fig 1, panel B). As the behavioural localization response necessarily takes some time to complete, one can argue that there is a further increase of the temporal window during which the memory for the vanishing location is being updated, thus critically determining the timeline that should be considered for any substantive estimation of the rate at which objects drifts downward in visual memory. The downward drift of the remembered vanishing location would, according to this view (see Fig 1, panel C–scenario 1), occur during the whole time window after the disappearance of the object until response completion. On the other hand, it might also be the case that the motor response is aimed directly at the perceived vanishing location at the end of the retention interval (see Fig 1, panel C–scenario 2). If the first scenario holds, it would be expected that, while the observer is performing the localization, further mnesic displacements downward would affect accordingly the motor trajectory and, thus, the path of the mouse cursor. For instance, it might be the case that mouse’s trajectory to curve downwards as its course is being corrected online with further mnesic distortions. Likewise, it would be expected that with longer times until response, the remembered location is further displaced downward. The second scenario, conversely, predicts that the motor response is made linearly and aimed at the location that is recalled at the end of the retention interval, with no course corrections made during response.

The main purpose of the present study was to ascertain which of those scenarios hold. In order to do so, participants were shown horizontally moving objects and required to locate, as precisely as possible, their vanishing positions on the screen after a variable retention interval. In order to induce variations in the time to complete each response, the gain of the computer mouse (ratio between spatial units of mouse displacement on the table and cursor displacement on the screen) was varied systematically. Although the exact relationship between the gain of the mouse and response times is not known, it was hypothesized that response times would vary monotonically with gain (with the lowest gain resulting in increased response times). Characterization of the times and dynamics of the motor responses using the mouse was thus set as a preliminary objective, for which the position of the cursor during each response was tracked approximately every 30 milliseconds. The data thus collected allowed the determination of the total response times, reaction time (defined as the moment during response when the cursor starts to be displaced), movement time (defined as the time during which the cursor is being displaced) as well as the spatial paths covered by the mouse cursor.

## Materials and Methods

### Participants

Ten participants (7 females; 3 males), with ages between 18 and 34 years (*M* = 27.9; *SD* = 5.1), volunteered for the experiment. All of them had normal or corrected to normal vision and were unaware of the purposes of the experiment. The experiment was approved by the local ethics committee of the Institute of Cognitive Psychology of the University of Coimbra and it was conducted in accordance with the Declaration of Helsinki and all participants provided written informed consent prior to the experiment.

### Stimuli

A set of animations portraying a black circle (*target*), with a diameter of 30 pixels (px; .9°), moving horizontally at a constant speed of about 477 px/s (14.3°/s) on an otherwise white background, were used as stimuli. The target’s trajectory was always shown centred vertically on the screen and the target could move rightward or leftward. The target vanished 90, 100 or 110 px (2.7°, 3° or 3.3°) after crossing the centre of the screen.

### Procedure and design

The experiment was run on a personal computer equipped with a flat screen with a resolution of 1280 × 1024 pixels (physical size of 33.7 × 27 cm; 37.2 × 30.2°) and a refresh rate of 60 Hz. Participants sat in front of the screen such that their cyclopean eye was aligned with the centre of the screen at about 50 cm. No head or eye constraints were imposed but participants were instructed to keep a steady posture during the entire experiment. Each participant completed 5 tasks, with the individual order determined by a Latin square design. For each task, only mouse sensitivity was varied using the *Windows* values 1, 3, 6, 11 and 20. Preliminary measurements with the apparatus used determined that those values resulted in gains (ratio between distance units on table and on the screen) of 0.25, 1.37, 4.02, 10.24 and 26.23. In all other respects, the task was exactly the same–the participants were required to observe the motion of the target and to remember its vanishing location. A retention interval of 0, 200, 400, 800, 1600 or 3200 ms between target offset and response initiation was imposed. The end of each retention interval was signalled by the appearance of a black circular cursor, with 5 px in diameter (0.15°), at the centre of the screen. The participants were required to position the cursor, using a computer mouse, at the location on the screen where they remembered the target vanishing, as precisely as possible and referring to its geometrical centre. The remembered location was confirmed with a single press on the left button of the mouse. The experiment was programmed in *Python* using the *PsychoPy* routines [30, 31], which recorded the location in pixels of each response as well as the response time. Moreover, a script was programmed in *AutoHotKey* which recorded the successive locations of the mouse’s cursor on the screen every 30 milliseconds. The experiment obeyed a full factorial repeated measures design given by 5 (task–mouse gain [blocked]) × 2 (motion direction) × 3 (vanishing position) × 6 (retention interval) with each stimulus combination being presented three times per participant.

## Results

Prior to the analysis, trials where response times were above 3 standard deviations above the mean of each condition or unusually low (< 200 ms) were discarded. Similarly, trials where participants made sudden circular random movements to identify the location of the cursor were eliminated. Globally, these amounted to about 1.5% of the total number of trials. For all performed analysis and dependent variables, motion direction failed to reach the statistical significance level (*F* < 1 in all cases) and is thus omitted in the remainder of the text.

### Temporal and spatial features of localization behaviour

**Fig 2:**
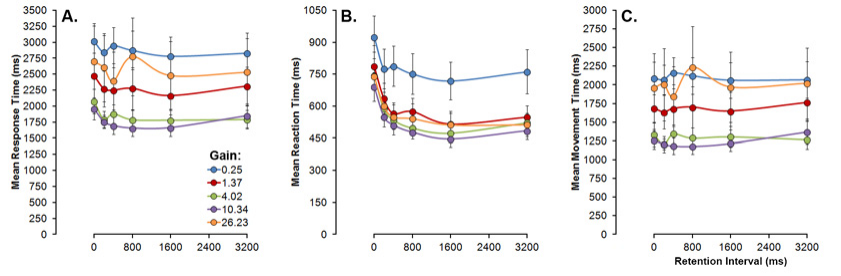
Mean response (Panel A), reaction (Panel B) and movement times (Panel C) as a function of retention intervals (abscissas) and gain conditions (line parameter). Vertical error bars depict the standard error of the means.

Response times were computed and averaged across replications and subjected to a repeated-measures ANOVA. Mean *response times* were found to be significantly increased for the 0 ms condition (see Fig 2, panel A), stabilizing for longer retention intervals, *F*(5, 45) = 6.16, *p* < .001, partial *η*^*2*^ = .41. Moreover, response times were significantly longer for vanishing positions further away from the centre of the screen, *F*(2, 18) = 3.77, *p* = .043, partial *η*^*2*^ = .29. Finally, changing the gain of the mouse resulted in significant differences in the response times (see Fig 2, panel A), *F*(1,47, 13.28) = 5.48, *p* = .025, partial *η*^*2*^ = .38, in a pattern were both the lowest and the highest gains resulted in increased times to complete the response. Arguably, the increased response times for the lower gains are due to the fact that longer arm movements are required to displace the cursor by the same amount. For higher gains, it might be the case that as more precise hand movements are required, participants take longer to respond to ensure that the cursor is not displaced by excessive amounts.

For each trial, the time until response initiation–*reaction time*–was computed individually based on the temporal delay between the end of retention interval and the moment when the cursor was first displaced from the centre of the screen (see Fig 2, panel B). The mean reaction times were found to be significantly higher for the 0 ms condition stabilizing for longer retention intervals, *F*(1.52, 13.67) = 15.42, *p* < .001, partial *η*^*2*^ = .63. Moreover, the lowest gain resulted in increased reaction times, *F*(2.1, 18.59) = 6.91, *p* = .005, partial *η*^*2*^ = .43.

*Movement time* was computed (see Fig 2, panel C) by subtracting the *reaction time* from *response time* for each trial and each participant. One ANOVA performed over the mean movement times revealed that only the gain of the mouse determined the time to complete the response movement, *F*(1.37, 12.35) = 4.84, *p* = .003, partial *η*^*2*^ = .59, with both higher and lower gains resulting in an increased time to complete the response movement. A post-hoc analysis (Bonferroni correction) revealed that the lowest gain condition (0.25) resulted in significantly higher movement times (*p* < 0.05) in comparison with the 4.02 and the 10.34 gain conditions, with no other contrasts reaching the statistical significance level.

In general, total response times seem to be decomposable on a short reaction time, affected mostly by the previous imposed retention interval (with the exception of the lowest gain, for which reaction times are also increased), and the time to complete the movement, highly modulated by proprieties of the apparatus such as the mouse gain.

**Fig 3:**
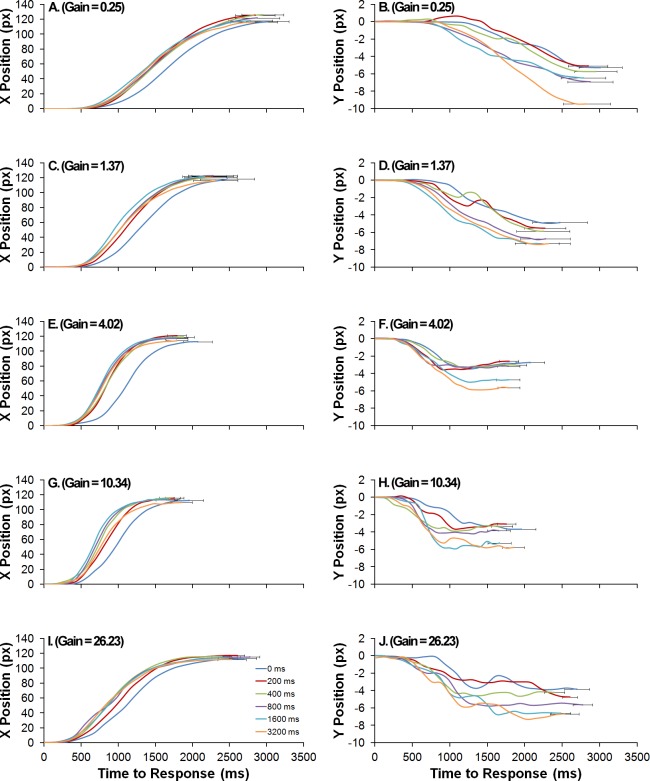
Mean horizontal (left column, panels A, C, E, G and I) and vertical (right column, panels B, D, F, H and J) positions of the cursor as a function of response time (abscissas) for the different gain conditions (rows) and retention intervals (line parameter). Horizontal error bars depict the standard error of the mean response times.

Fig 3 depicts the mean horizontal (left column) and vertical (right column) locations of the cursor as a function of response time for the different gain conditions (rows) and retention intervals (line parameter). It can be seen that after the end of the retention interval, the cursor is kept for some time at the centre of the screen and that this delay in response initiation seems somewhat longer for the 0 ms retention interval, in accordance with the increased reaction times found for this condition. After this brief temporal interval, cursor’s horizontal position changes increasingly rapidly, slowing its rate of change (slope of the lines) when approaching the desired and final location. The last few hundred milliseconds comport minor adjustments of the mouse cursor which, in the averaged data shown in Fig 3, result in a slight plateau. Varying the gain of the mouse seems to have little effect on the overall patterns for the horizontal displacement of the cursor (except on what refers to response times), but it seems to affect considerably the dynamics of the vertical component. For the lowest gain, the vertical position of the cursor increases steadily downward as the response unfolds until reaching the desired location. With increases in mouse gain, the desired vertical location seems to be achieved systematically sooner. In particular, for the 4.02, 10.34 and 26.23 gain conditions, by the time the behavioural response is still midway its complete trajectory, the vertical displacement of the cursor achieves its lowest and final position, with only some small adjustments made until response, noticeable at the individual level.

**Fig 4:**
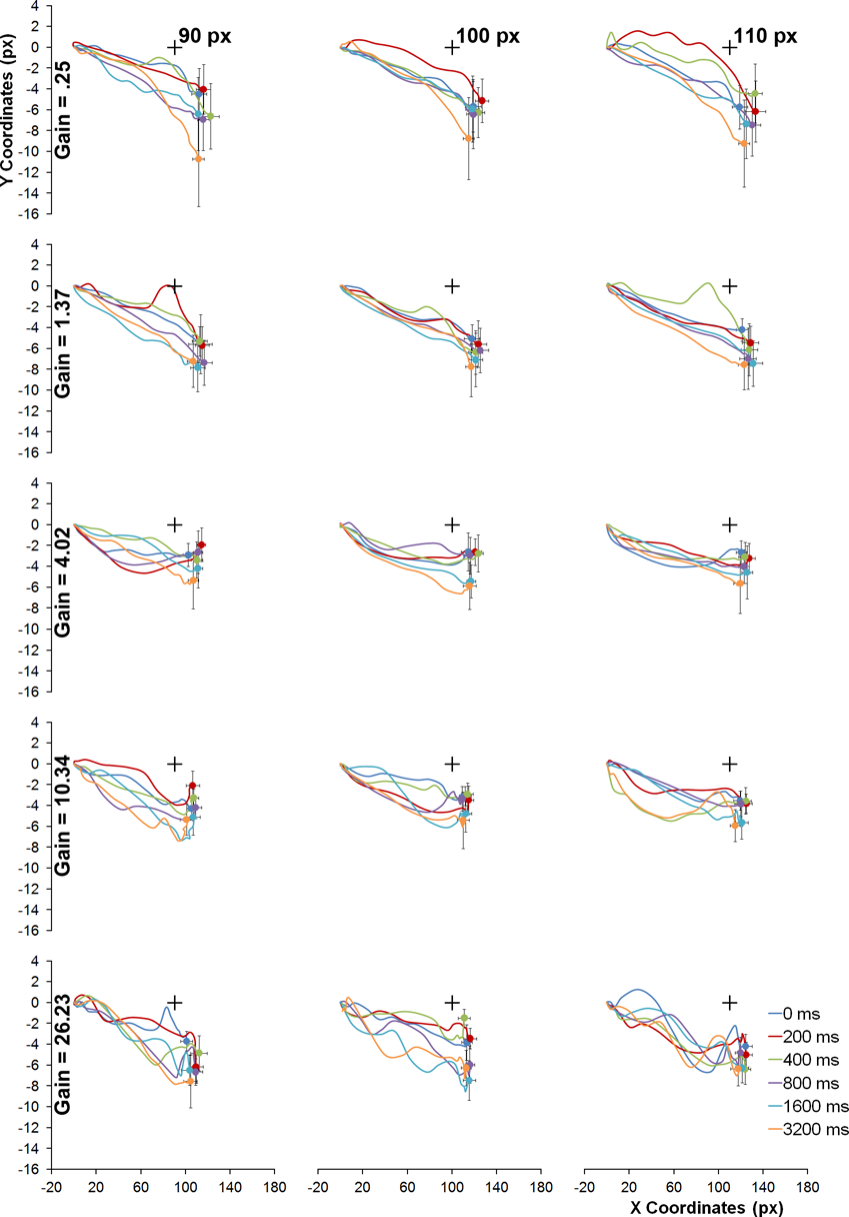
Mean spatial trajectories of the mouse’s cursor made during response for the different gain conditions (rows), vanishing locations (columns and black crosses) and retention intervals (line parameter). Data was computed such that positive numbers refer to a movement made toward the vanishing location and averaged across the leftward and rightward motion conditions. Error bars depict the vertical and horizontal standard errors of the mean judged locations.

Fig 4 depicts the mean spatial trajectories of the mouse, from the centre of the screen to the remembered location, for different gain conditions (row panels), actual vanishing location (black cross; column panels) and retention interval (line parameters). Notice that for the 0.25 gain condition, which resulted in increased movement times, there is a slight trend for the spatial trajectories of the mouse to be curved downwards, while for the 4.02 and 10.34 conditions the inverse seems to hold. For the remaining conditions, the trajectories seem somewhat linear.

In order to further explore the spatial trajectories of the mouse at the individual level, a quadratic function was fitted to the successive horizontal and vertical locations of the cursor for each trial, rescaled to a common absolute distance (for a similar procedure applied to saccade curvature see [32]). The coefficients of the quadratic term index any significant curvature of the mouse’s spatial trajectory, with a negative coefficient corresponding to a downward curvature.

One ANOVA performed over the individual mean quadratic coefficients revealed that no factor significantly determined the response movement’s curvature: mouse’s gain, *F*(1.15, 10.39) = 1.58, *p* = .201; retention interval, *F*(1.04, 9.35) < 1; motion direction, *F*(1, 9) < 1; vanishing location, *F*(1.01, 9.06) < 1. Moreover, the mean quadratic coefficients were not significantly different from 0, *t*(9) = 1.75, *p* = .115, two-tailed. As movement times were found to differ only between the 0.25 and both the 4.02 and 10.34 gain conditions, a second ANOVA performed on the quadratic coefficients for only these conditions revealed a significant effect of gain on response curvatures, *F*(2, 16) = 3.8, *p* = .045, partial *η*^*2*^ = .6 (all the remaining factors resulted in a *F* < 1), although not in the direction suggested by visual inspection: for the lowest gain, the quadratic coefficients were not significantly different from 0 (*p* > 0.05); for the 4.02 and 10.34, the trajectories were significantly curved upwards. This finding is in line with the temporal pattern disclosed in Fig 3 for the vertical displacement of the mouse’s cursor. It is noteworthy that the spatio-temporal unfolding of the behavioural response in the 4.02 and 10.34 resembles an intersection action, with its vertical component being rapidly adjusted to be below the actual vanishing location of the target, allegedly disclosing a mnesic displacement downward. In that sense, that the lowest gain (0.25) changes this pattern might be taken to reflect the functioning of an on-line spatial updating of the object’s location.

Be the case as it may, the analysis performed so far suggests that the 0.25 gain was effective in inducing increased response times and disparate spatial and temporal response dynamics, as compared with the 4.02 and 10.34 conditions, and should thus be the main focus for exploring the degree to which the remembered vanishing locations vary with response times.

### Spatial localization errors

For each trial, the horizontal difference in pixels between the location indicated by the participant and the actual vanishing position was calculated so as to obtain the magnitude of the errors along the target’s motion axis–M-displacement. Notice that positive M-displacement values refer to errors made forward in the direction of motion (see Fig 1, panel B). Likewise, the vertical errors in pixels were calculated so as to obtain the magnitude of displacements orthogonal to the direction of motion–O-displacement. Negative O-displacements index an error made downward in the direction of gravity (see Fig 1, panel B). The data thus obtained was averaged across replications and subjected to two repeated-measures ANOVAs, one for each error component (M- and O-displacement). Whenever the sphericity assumption was not met the Greehouse-Geisser correction for the degrees of freedom was performed.

**Fig 5:**
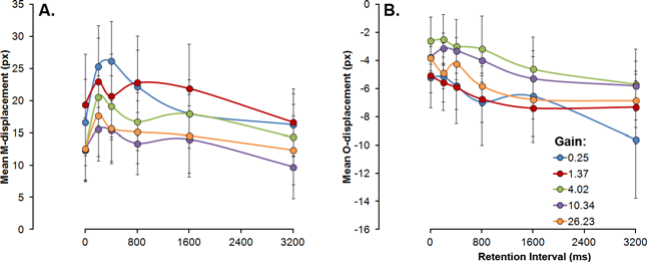
Mean M- (Panel A) and O-displacements (Panel B) in pixels as a function of retention intervals in milliseconds. Vertical error bars depict the standard error of the means.

For M-displacement, significant effects of mouse gain, *F*(4, 36) = 3.57, *p* = .015, partial *η*^*2*^ = .28, retention interval, *F*(5, 45) = 5.97, *p* < .001, partial *η*^*2*^ = .39, and vanishing location, *F*(2, 18) = 26.02, *p* < .001, partial *η*^*2*^ = .74, were found. No other factor or interaction reached the statistical significance level. Overall, M-displacement decreased with both increases in the mouse gain (see Fig 5, panel A) and vanishing locations further beyond the centre of the screen. As for retention interval, M-displacement was found to increase until a peak at about 200–400 ms and to decrease steadily for longer retention intervals (see Fig 5, panel A).

As for O-displacement, only retention interval was found to modulate its magnitude, *F*(1.29, 11.69) = 5.04, *p* = .038, partial *η*^*2*^ = .36. Mouse gain had neither a principal effect on O-displacement, *F*(4, 36) = 1.127, *p* = .359, nor did it interact with other variables. In general, O-displacement increased steadily downward with increases in the retention interval (see Fig 5, panel B).

In order to inspect the relationship between O-displacement and total time until response, and since movement times were found to vary in a non monotonic relation in respect to mouse’s gain, mean O-displacements were calculated for the 0.25 –*long movement times–*and for the aggregation of the 4.02 and 10.34 –*short movement times–*gain conditions. A repeated-measures ANOVA was performed on the mean O-displacements with movement times (*short* or *long*), target’s covered distance (also found to significantly increase movement times) and retention interval as factors.

**Fig 6:**
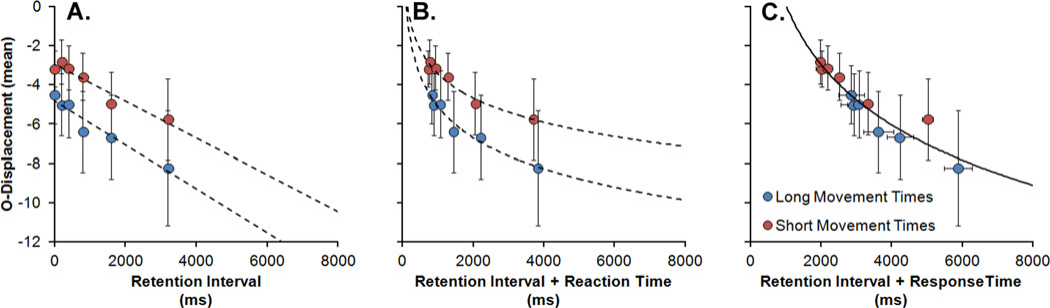
Mean O-displacement as a function of retention interval (Panel A), retention interval plus reaction (Panel B) and response times (Panel C) for the conditions with *short* (red data points) and *long* (blue data points) movement times. Vertical and horizontal error bars depict the standard error of the mean for O-displacements and response times, respectively.

With increases in retention interval, O-displacement was found once again to increase downwards, *F*(1.37, 12.37) = 4.86, *p* = .038, partial *η*^*2*^ = .6. Target’s covered distance did not reach the statistical significance level, *F*(2, 18) = 2.41, *p* = .118. Finally, movement time was found to have a marginally significant effect, *F*(1, 9) = 4.15, *p* = .072, partial *η*^*2*^ = .45, with longer times to response completion resulting in localizations slightly more displaced downwards (see Fig 6, panel A). Despite the fact that this difference did not reach the statistical significance level, it does suggest that the spatial updating of the remembered vanishing location might be extended beyond the imposed retention intervals. Panel B of Fig 6 depicts the mean O-displacement values for both the *short* and *long* retention intervals as a function of the sum of retention intervals and reaction times (averaged for each corresponding condition). It can be seen that considering reaction times does introduce a slight non-linearity on the data points, due to the fact that participants took longer to react for the shorter retention intervals. Notwithstanding, the difference in O-displacement between the *short* and *long* movement times does not seem to be significantly reduced. When the total time until response is, however, considered (Fig 6, panel C), the two sets of data do seem to lie in a common negatively accelerated function, suggesting that the increased time might account for the small difference in O-displacements. In favour of this hypothesis, a logarithm function (with two free parameters) was found to fit the O-displacement data points in Fig 6 (*SSE* = 3.13, *R*^*2*^ = .89, *RMSE* = .56) significantly better for retention intervals plus response times in comparison with retention intervals plus reaction times, *F*(10, 10) = 3.5, *p* = .03, as well as in comparison with a linear function for retention intervals alone, *F*(10, 10) = 4.47, *p* = .013.

## Discussion and Conclusion

**Fig 7:**
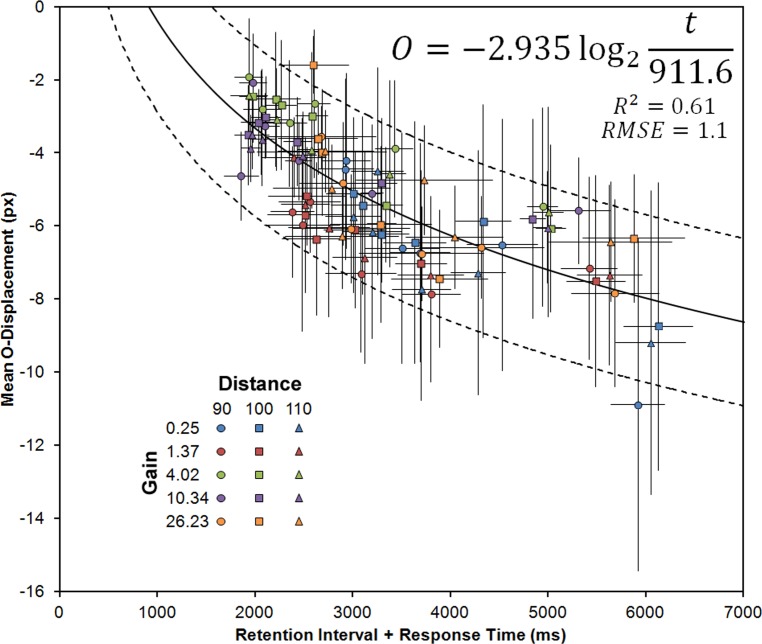
Mean O-displacement plotted as a function of retention plus response times. Coloured data points correspond to different gain conditions and covered distances (different shapes). The continuous line depicts the best fit logarithm function, given by the accompanying equation, and the dashed lines the 95% prediction bounds (computed with MATLAB). Error bars depict the standard error of the mean for O-displacement (vertical bars) and response time (horizontal bars).

The outcomes reported on the present paper fully support the previous findings wherein the remembered vanishing location of moving targets drift downward, in the direction of gravity, with time and as if following an anticipated trajectory [26]. Of importance, the present study provides two important outcomes that further clarify the relationship between time and downward displacement with this sort of behavioural localization tasks. On the one hand, evidence was found that, depending on the extension of the imposed retention interval, participants might take longer to initiate their motor behaviour [17, 27]. Given that the task explicitly requires participants to wait for the cursor appearance before responding, and as spatial precision is encouraged instead of a fast response, it is plausible to assume that when the cursor appears immediately after the target’s disappearance (as in the 0 ms condition) the motor response is not yet planned, resulting in increased reaction times. On the other hand, the found outcomes lend some credence to the hypothesis that the spatial updating of the remembered vanishing location extends in time until a response is provided. That is, the visual memory for the object’s location seems to drift downward beyond the imposed retention interval, extending during at least part of the time it takes for the observer to provide a response. It should be noticed, however, that this conclusion stems from an effect that was found to be only marginally significant. To some extent, it might be the case that, as mouse’s gain was manipulated with a blocked design, participants attempted to adjust their motor behaviour in order to cope with the different device’s sensitivities, thus equalizing their movement times. This fact alone might have contributed, more than expected, to a decrement in statistical power. Moreover, analysis of the behavioural movement trajectories, although affected by the manipulation of mouse’s gain, failed to provide a clear support for either the existence or absence of an online spatial updating. For the lowest gain condition, which resulted in increased response times, some slight evidence of a downward curving trend was observable, which was not confirmed with statistical analysis, suggesting that a linear movement was performed on several trials. For the 4.02 and 10.34 gain conditions, which lead to significantly shorter response times, the vertical position of the mouse cursor increased downward almost linearly, stabilizing at the final position in about half the total time until response (while the horizontal position was still being adjusted). The most parsimonious account of the found outcomes is that the remembered vanishing location was being updated online but only until a match was reached between the internal generated spatial location estimate and the mouse’s cursor position. In all cases, that the mean O-displacements were found to cluster around a common function when the sum of retention and response times are considered, provides convincingly evidence that the mechanisms responsible for representational gravity do extend further in time until a response is provided. Fig 7 depicts O-displacement values plotted against time until response (retention intervals plus response times) for the different gain conditions (data points’ colours) and object’s covered distances (data points’ shapes), both found to affect response times. In line with the reported findings, the data points seem to lie on a single negatively accelerated function, here tentatively approximated with the following equation:

$$O=a\;{\text{log}}_{2}\frac{t}{t_{0}}$$ (1)

Parameter *a* indexes the rate at which the remembered vanishing location, given by *O*, drifts downward as a function of time–*t*. Parameter *t*_*0*_ provides an estimate of the time at which O-displacement equals 0. The best fit (*R*^*2*^ = 0.615, *SSE* = 108.7, *RMSE* = 1.11) was found for an *a* of about -2.935, 95% CI [-2.443; -3.427], and *t*_*0*_ of about 911.6, 95% CI [716.8; 1107]. These values suggest that (i) O-displacement increases downward by about 3 pixels for each two-fold increase in time, and (ii) there seems to be no O-displacement until about 911 milliseconds after target’s offset. As observers necessarily take some time to respond, it is not possible with the present set of data to ascertain if indeed O-displacement takes this long to emerge–it might be the case that the value of *t*_*0*_ is but an artefact of behaviour localization responses. However, taking this value at face value do suggests that the remembered vertical vanishing location is somewhat accurate during the first few hundred milliseconds after object’s offset. Interestingly, this temporal frame would posit representational gravity at a processing stage beyond the so-called “iconic memory”, thought to be a highly accurate albeit fast decaying buffer of visual inputs [33, 34, 35]. Specifically, it would plausibly suggest that the spatial updating of the object’s location, as well as the functioning of an internal model of gravity, starts sensibly at the moment when visual working memory is recruited to the task–the time range is certainly compatible with reports from the relevant literature [36, 37]. One way to address this issue would be to assess memory displacements downward for shorter times, eventually using a probe methodology [11], although the direct comparison between these two disparate methodologies is not without difficulties (cf., e.g., [13]). Irrespective of the ultimate answer to these questions, the present outcomes highlight that, even for studies in which the timing of mnesic displacements is not being explicitly considered, temporal factors (in the form of response times) do certainly play a role that should not be ignored.

For instance, that increased response times contribute to the time during which the remembered vanishing location drifts downward sheds some light on a previous report [26] where O-displacement was found to increase downward at a seemingly faster rate for short retention intervals (about 7.5 pixels per second for retentions between 0 and 300 ms) as compared to longer intervals (about 3.4 pixels per second for retentions between 0 and 1,200 ms) irrespectively of eye movements’ constraints. Interestingly, O-displacements differed for equal retention intervals in those cases where long and short retention intervals overlapped (0 and 200 ms). It might be surmised, in accordance with the present findings, that in an experimental context where participants are confronted with generally longer retentions, their reactions times, especially for the lower intervals, is somewhat increased due to heightened expectations for a longer delay, which would explain the apparent discrepancy. Arguably, if response times were considered in that previous report, the found O-displacements would lie in a single negatively accelerated function, with memory for the position of the target rapidly drifting downward with time at first and stabilizing afterward.

In any case, the outcomes found in the present report fully support that memory for the vanishing location of a moving target drifts downwards with time–representational trajectory–thus providing further evidence favouring that this sort of spatial updating tackles a predictive internal model of gravity. Of relevance, and altogether, the present findings are more favourable than not to the hypothesis that this internal model iteratively outputs a localization estimate even during a motor behavioural action, which might thus be corrected on-line.

Importantly, changing the gain of the mouse significantly affected the mnesic displacements forward in the direction of motion, where increases in mouse gain lead to decreases in M-displacement. Since motor response times are not monotonically related with mouse gain, as both the biggest and smallest gains lead to increased response times, one can confidently dismiss a role of temporal variables in M-displacement. Instead, changes in M-displacement magnitude seem to be related with biomechanical variables such as the amplitude and dynamics of required arm movements. Although no full account of this effect can be provided at the moment, it is worth to notice that M-displacement has been reported, likewise, to be modulated by eye movements (cf. e.g., [26]). It might be the case that a link exists between oculomotor dynamics and arm movements, although its exact nature is still far to be guessed upon.

As a final remark, notice that with longer times until response completion, the vertical spread of the locations indicated by the observers increase, as evidenced by the standard errors of the means (bars in Figs 6 and 7). This trend provides support for our recent proposal that the functioning of the neural mechanism responsible for the spatial updating of the target’s location might be akin to Kalman filtering [28]–as the visual input decays after target’s disappearance the perceptual contribution to the remembered location becomes noisier, leading the observer to rely increasingly more on the predictions generated by an internal model of gravity. The findings reported in the present paper offer important insights on the dynamics of such mechanism and contribute to constrain possible computational implementations.
